# Metabolomics reveals an involvement of pantothenate for male production responding to the short-day stimulus in the water flea, *Daphnia pulex*

**DOI:** 10.1038/srep25125

**Published:** 2016-04-26

**Authors:** Kenji Toyota, Alex Gavin, Shinichi Miyagawa, Mark R. Viant, Taisen Iguchi

**Affiliations:** 1 Okazaki Institute for Integrative Bioscience, National Institute for Basic Biology, National Institutes of Natural Sciences, and Department of Basic Biology, Faculty of Life Science, SOKENDAI (The Graduate University for Advanced Studies), 5-1 Higashiyama, Myodaiji, Okazaki, Aichi 444-8787, Japan; 2 Environmental Genomics Group, School of Biosciences, University of Birmingham, Edgbaston, Birmingham B15 2TT, U.K; 3 Environmental Metabolomics Research Group, School of Biosciences, University of Birmingham, Edgbaston, Birmingham B15 2TT, U.K

## Abstract

Under favorable conditions, the micro-crustacean *Daphnia pulex* produces female offspring by parthenogenesis, whereas under unfavorable conditions, they produce male offspring to induce sexual reproduction (environmental sex determination: ESD). We recently established a suitable system for ESD studies using *D. pulex* WTN6 strain, in which the sex of the offspring can be regulated by alterations in day-length; long-day and short-day conditions can induce female and male offspring, respectively. Taking advantage of this system, we have already demonstrated that methyl farnesoate (MF) synthesis is necessary for male offspring production, and identified ionotropic glutamate receptors as an upstream regulator of MF signaling. Despite these findings, the molecular mechanisms associated with MF signaling have not yet been well elucidated. In this study, we analyzed the whole metabolic profiles of mother daphnids reared under long-day (female-producing) and short-day (male-producing) conditions, and discovered that pantothenate (vitamin B5), a known precursor to coenzyme A, was significantly accumulated in response to the short-day condition. To confirm the innate role of pantothenate in *D. pulex*, this metabolite was administered to mother daphnids resulting in a significantly increased proportion of male offspring producing mothers. This study provides novel insights of the metabolic mechanisms of the ESD system in *D. pulex*.

The water flea genus *Daphnia* is a representative zooplanktonic crustacean ubiquitously found in freshwater ponds and lakes around the world, and has a critical role as a primary consumer in the food chain. A trait common to all *Daphnia* species is their cyclical parthenogenetic reproductive cycle, in which parthenogenesis and sexual reproduction can be altered in response to various environmental cues such as photoperiod, temperature, nutrients, crowding and combinations of these stimuli^1^,^2^,^3^,^4^. During favorable conditions for growth, offspring are produced parthenogenetically, resulting in a population consisting of genetically identical females. When unfavorable conditions (e.g., shortened day length, low temperature, lack of nutrients and crowding) arise, genetically identical male offspring are produced to induce sexual reproduction in the population resulting in the production of resting eggs. In this way, daphnids take advantage of cyclical parthenogenesis depending on environmental conditions; during the favorable season, parthenogenesis allows rapid propagation, while sexual reproduction contributes to an increase in genetic diversity and survival rate in changeable environmental conditions^5^. Therefore, the trigger of sexual fate determination and its regulating molecular pathways are fundamental mechanisms to be elucidated in order to better understand daphnid reproduction and ecology in seasonally changing environments^3^.

In spite of large efforts, however, the induction system for male offspring has not yet been established^1^,^2^,^3^,^4^. This is because multiple environmental cues (*e.g.*, short-day length, low temperature, oligotrophy and overcrowding) appear to be integrally involved in daphnid sex determination, thereby making it difficult to reproduce such experimental conditions in the laboratory. Recently, several groups including our own have reported that exogenous administration of juvenile hormones (JHs: representative endocrine factors among arthropods) or their analogs can induce the production of male offspring even under female-producing conditions^6^,^7^,^8^,^9^. The contribution of endogenous JH signaling on male-fate determination, however, has so far been elusive due to the lack of a suitable experimental system which allows the modification of offspring sex without the use of exogenous JH. To overcome this limitation, we have recently established a reliable system for the induction of both female and male offspring in *D. pulex* strain WTN6 through alteration of culture day-length. A mother produces female neonates when reared under the long-day condition (14 h light : 10 h dark), while under the short-day condition (10 h light : 14 h dark), male neonate production is induced^10^. Using this male offspring induction system, we have recently demonstrated that methyl farnesoate (MF) is likely an innate JH in daphnids^10^. Moreover, using transcriptome analysis, we revealed that *N*-methyl-D-aspartic acid (NMDA) receptors (a type of ionotropic glutamate receptor) are an important factor for male offspring production in *D. pulex* WTN6 strain acting as an upstream regulator of MF signaling^11^.

Omics technologies enable us to analyze alterations of an organism’s global molecular profile in response to changes in environmental conditions. An organism’s metabolic profile can be considered as the manifestation of gene expression and cellular metabolism, defined as the metabolic phenotype^12^. Recent omics studies using *Daphnia* species have shown that a combination of transcriptional and metabolic profiles enables a more complete understanding of the molecular level response to a stress^13^. Consequently, the coupling of our existing transcriptomics data with metabolomics data should enable the mapping of the initial transcriptional changes onto the phenotypic response of *D. pulex* to the short-day conditions.

Our first objective in this study was to further investigate the factors involved in sex determination during the critical period. To achieve this, we performed direct infusion mass spectrometry (DIMS) based metabolomics using the adult *D. pulex* WTN6 strain under female-inducing (long-day) and male-inducing (short-day) conditions; i.e., to attempt to discover candidate metabolites in the mother daphnids that are associated with the production of male offspring. Our second objective was to use pharmacological manipulation to validate these candidate metabolite(s) as being capable of inducing male offspring in *D. pulex* WTN6 strain. As such, we sought to contribute towards the understanding of how organisms translate external environmental information into biological information.

## Results

### Differential metabolic profile in response to short-day conditions

Our initial aim was to discover differential levels of metabolites between female- and male-producing mothers. The *D. pulex* WTN6 strain can switch the sexual fate of its offspring depending on day-length conditions^10^. Under long-day conditions, they produce 100% female offspring, while under short-day length they produce predominantly male offspring^10^,^11^,^14^. In detail, WTN6 strain produces female and male until one-month age (5 clutches), and then, they produce approximately 100% male offspring. Our experiment used only one-month age daphnids, enabling the comparison with mother reared under 100% female- or male-producing conditions^10^. Prior to the metabolomics investigation, we evaluated whether the two rearing conditions used in this study impacted the growth of *D. pulex* through a comparison of body length, as such an effect would likely be accompanied by metabolic changes that could complicate the interpretation of the metabolomics data. Body length measurements showed no significant difference between the day-length conditions (Fig. S1), indicating that this experimental design was suitable for searching for the metabolic factors that regulate the physiological changes of mother daphnids.

**Figure 1:**
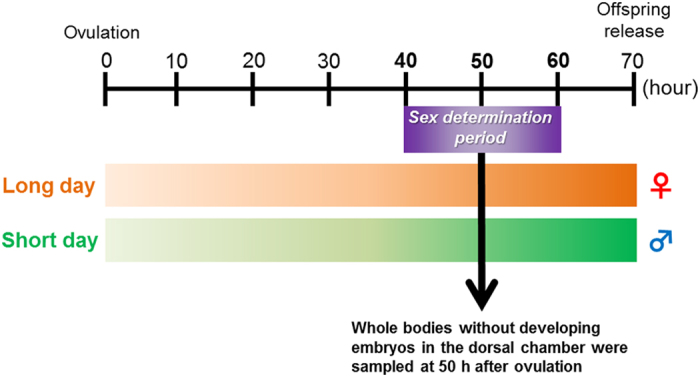
Experimental design for the metabolomics analysis. The illustration shows the reproductive cycle (approximately 70 hours/cycle under 18 °C) in *D. pulex* WTN6 strain and the sampling times for this study. The space between the dotted purple lines indicates the methyl farnesoate (MF)-sensitive period for male offspring production by exogenous MF treatment (40–60 h after ovulation). At 50 h after ovulation, all daphnids were sacrificed and prepared for metabolomics analysis.

**Figure 2:**
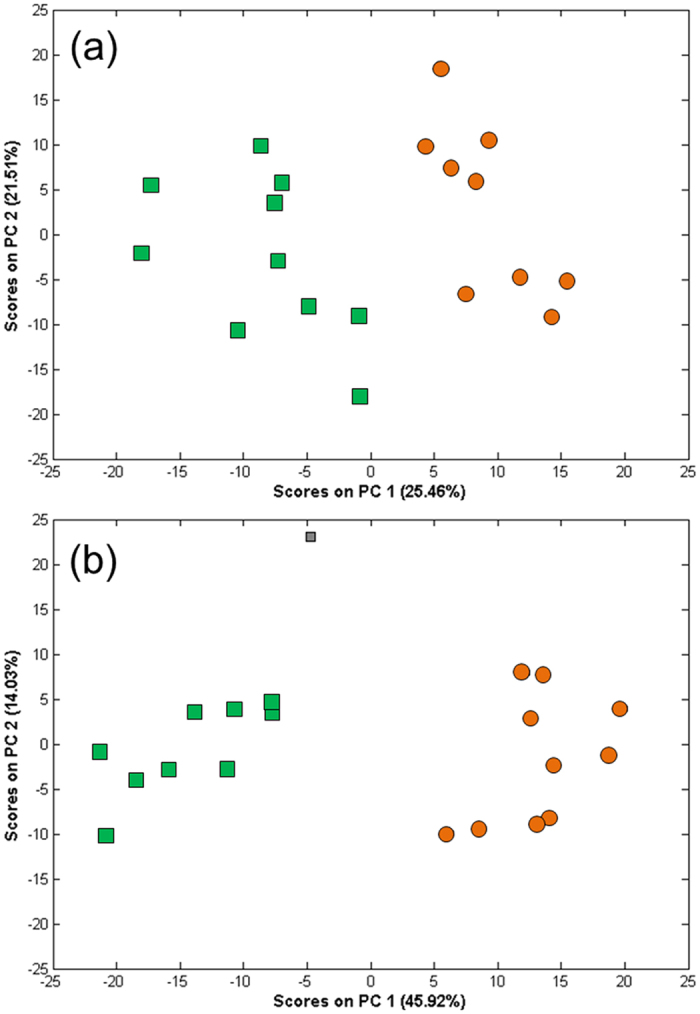
PCA scores plots of positive (**a**) and negative (**b**) ion mode mass spectra describing the metabolomes of the *D. pulex* WTN6 strain following long-day (orange ⦁) and short-day (green ▪) culture conditions. The differences between the PC1 scores of the two groups were determined to be significant by *t*-test (*p* < 0.0001) in both ion modes.

DIMS based metabolomics analysis was conducted on mothers reared under long-day (female-producing) and short-day (male-producing) conditions (Fig. 1). Following data acquisition and spectral processing, PCA was used to visualize the metabolic differences between all of the animals for data acquired in both positive and negative ion modes. A clear separation was observed between animals cultured under the long-day and short-day conditions along PC1, for both ion modes (*t*-test of PC1 scores, *p* < 0.0001; Fig. 2). To discover the peaks responsible for this separation, the PC1 loadings were reviewed, and in addition *t*-tests (with FDR correction) were applied between the two culture conditions to identify those peaks that differed significantly in intensity. In positive ion mode, 265 (of 1905) *m/z* peaks differed significantly, while 731 (of 1744) occurred in negative ion (*q* < 0.05). Of the top 200 peaks with the largest PC1 loadings (specifically, largest absolute values of the loadings data) in each PCA model, 74.5% and 89.5% changed significantly in intensity in positive and negative ion modes, respectively, confirming that the two lists of significantly changing peaks accurately reflect the changes observed in the PCA scores plots.

### Functional annotation of accumulated metabolites in response to the short-day stimulus

In an attempt to annotate those mass spectral peaks that differ significantly between the long-day and short-day conditions, empirical formula(e) were assigned using MI-Pack software. Next, utilizing the KEGG database, the transformation mapping algorithm in MI-Pack was used to assign putative metabolite names to these empirical formulae^15^. In total, 112 *m/z* peaks that increased in intensity and 78 peaks that decreased in intensity, under short-day conditions, were assigned at least one putative compound name from KEGG (Tables S1 and S2, respectively). Under the short-day conditions, these putatively annotated metabolites were between *ca.* 6-fold higher in intensity to *ca.* 3-fold lower, compared to the animals reared under the long-day conditions.

**Figure 3:**
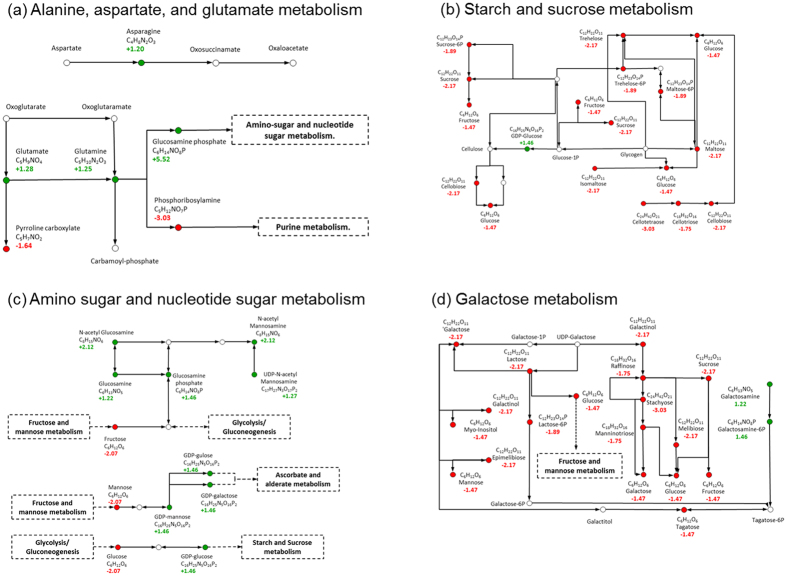
Pathway maps for the alanine, aspartate and glutamate metabolism (**a**), starch and sucrose metabolism (**b**), amino sugar and nucleotide sugar metabolism (**c**), and galactose metabolism (**d**), adapted from the respective pathways in the KEGG database. Pathways maps are limited to reactions involving putatively annotated compound names associated with significantly increasing (green ⦁) or decreasing (red ⦁) *m/z* peaks in response to the short-day conditions. The positive or negative fold change of the most stable detected *m/z* ion form is displayed for each putative compound name. Compounds that were not detected, but which are known to be involved in these reaction pathways, are also included (⚬). Solid black lines represent interactions between pathway components. Dashed lines are included when a particular metabolite is also included in another KEGG pathway.

A list of all putative KEGG IDs was input into MetaboAnalyst^16^ and over-representation metabolic pathway analysis was conducted. Following FDR correction for multiple testing^17^, no KEGG pathway definitions were found to be significantly enriched (Table S3). To highlight those pathway definitions of potential interest, a filter was implemented to retain only those with 6 or more significant putatively annotated metabolites. This resulted in 18 KEGG pathway definitions potentially implicated in the production of male offspring. The putative constituents of the alanine, aspartate, and glutamate metabolism pathway tended to increase under male offspring producing conditions (Fig. 3a). Moreover, when ranked by uncorrected *p*-value, three of the top five pathways show consistency and are involved in the metabolism of sugars. This includes starch and sucrose metabolism (Fig. 3b), amino sugar and nucleotide sugar metabolism (Fig. 3c), and galactose metabolism (Fig. 3d). All of these pathways show significant decreases in levels of putatively annotated mono- and oligosaccharides, but increases in the levels of amino-sugars, sugar phosphates and nucleotide-sugars (Fig. 3c). These changes are observed consistently across all of the ion forms of each of these putatively identified metabolites (Tables S1 and S2).

### Administration of pantothenate promotes male production in WTN6 strain under the female-producing conditions

**Figure 4:**
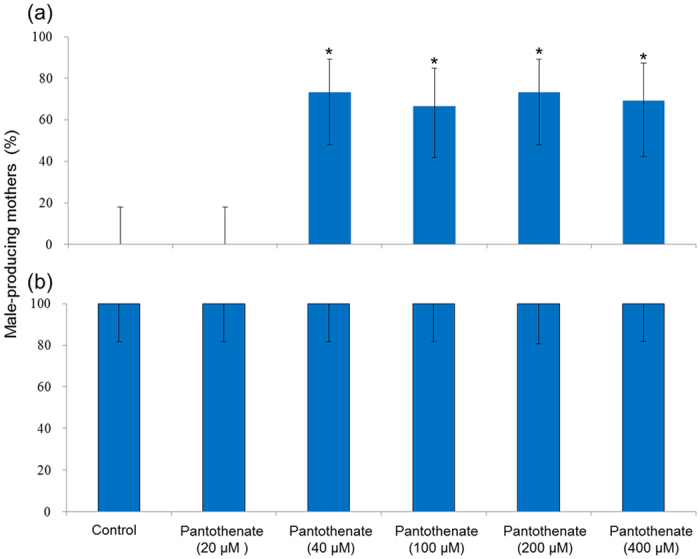
Effects of pantothenate on the inducibility of male offspring by mothers reared under the long-day (**a**) and short-day (**b**) conditions. Data are presented as the percentage (%) of male-producing mothers (n = 13–15). Bars indicate the 95% confidence interval. The asterisks indicate significant differences compared to controls (Fisher’s exact probability test with Holm’s correction, *p* < 0.01).

Further investigation of the significant putatively annotated peaks revealed that the peak with the highest absolute fold change in intensity between culture conditions (5.9 times higher in short-day cultured *Daphnia*) had the putative annotations of pantothenic acid (vitamin B5), N-acetyl-D-glucosamine, N-acetyl-D-mannosamine and (3-(acetylhydroxyamino)propyl) phosphonic acid (Table 1). Of these putative identities, pantothenic acid has been previously documented to promote egg production in *D. pulex*^18^. To investigate the molecular functions of pantothenate and its potential contribution to male induction, we performed direct exposures of the *D. pulex* to this metabolite. First, we exposed mothers reared under the long-day condition to a range of concentrations of pantothenate and discovered that the percentage of male-producing mothers increased in response to the exposure (Fig. 4a). In contrast, administration of pantothenate did not affect the proportion of male-producing mothers reared under the short-day condition (Fig. 4b). In these experiments, the individual mothers always produced either female or male offspring in a clutch.

## Discussion

Our metabolome analysis showed that several components of the alanine, aspartate, and glutamate metabolism pathway seemed to accumulate under the male-producing conditions. In particular, an increased level of glutamate observed in this study is well consistent with our previous transcriptome data^11^, which showed ionotropic glutamate receptor-related genes were significantly changed in response to the short-day conditions, and specific agonists/antagonists of these receptors induced/inhibited the male production, respectively^11^. Although these data suggest that the glutamate might act as an innate ligand of ionotropic glutamate receptors, further study will be required. These findings provide important clues about the molecular signaling network regulating male offspring production, especially the involvement of glutamate signaling pathways in the ESD system of the WTN6 strain of *D. pulex*.

Since the body lengths of daphnids cultured under the two conditions were not significantly different, this suggests that the reduction in the levels of putative mono- and oligosaccharides is unlikely to result from starvation or malnutrition induced indirectly by the short-day condition. One potential cause could be the increase of polysaccharide (glycogen) synthesis and sugar storage which may rapidly consume the simple sugars, leading to decreases in their peak intensities. Peaks assigned putative identities of phosphorylated monosaccharides and nucleotide sugars increased significantly in response to the short-day culture conditions. Both of these families of compounds are crucial intermediates in the synthesis of glycogen^19^,^20^,^21^. As a complex polysaccharide consisting of multiple combined sugar-units, glycogen itself will not be observed in the 70–590 *m/z* range in this study. However, the increased intensities of putative precursors may allude to an increase in its production. Interestingly, our previous transcriptome analysis revealed that expression levels of genes involved in the amino sugar metabolic process (GO: 0006040), glucosamine-containing compound metabolic process (GO: 1901071), and aminoglycan metabolic process (GO: 0006030) were changed in mothers reared under the long-day with MF-treatment (*i.e.*, were male-producing)^11^. Although these data indicate that glycogen synthesis processes might be regulated downstream of the MF signaling pathway, a causal relationship between those metabolite pathways and the regulatory mechanism of male offspring production remains largely unknown.

Pantothenate is ubiquitously present in all living organisms, a known precursor of co-enzyme A (CoA) and is also a constituent of the β-alanine metabolism pathway; one of the 18 pathways of interest following analysis of the daphnid metabolome described above. It has been previously documented as promoting egg production in *D. pulex*^18^. Our metabolome analysis demonstrated that pantothenate levels were increased in response to male-producing conditions. Two possibilities about difference results of pantothenate effects between previous and current studies; one is healthy (culturing) conditions of daphnids, the other is species difference. In the former case, average number of producing offspring was about 6 and more than 30 individuals in previous and current study under the non-pantothenate treatment group, respectively, suggesting that our daphnia culturing conditions seem to reach maximum fecundity. Therefore, we considered that we could not observe the increase of offspring number in response to pantothenate treatment. In the latter case, *Daphnia magna* and *D. pulex* were used in previous and current our study, respectively. Therefore, it might be caused by species difference, although further study will be required to clarify whether this pantothenate function as a male inducer is conserved among cladocera species. Moreover, pharmacological treatment with pantothenate induced male offspring production even under female-producing conditions. Administration of JH agonists to daphnids at the critical sex-determining (oocyte-maturing) period has previously been shown to induce male offspring production^10^,^22^,^23^. However, in order to induce male offspring production by pantothenate exposure, the administration needed to occur before the critical sex-determining period (see Materials and methods). We observed all produced offspring number and their sexes during pantothenate exposure. If pantothenate directly affected embryos in mother’s brood chamber, all embryos in the brood chamber when pantothenate treated to mother become the male. However, we revealed that all embryos became female (data not shown). These data suggested that pantothenate affects mother daphnids and alters offspring sexual fate into male. Taken together, the current results suggest that pantothenate acts upstream of MF signaling for male offspring production in the *D. pulex* WTN6 strain.

The underlying mechanism that connects the physiological role of pantothenate and the activation of MF signaling remain largely unclear. One possibility is that pantothenate acts as primary source for the MF biosynthesis pathway. In the budding yeast *Saccharomyces cerevisiae*, pantothenate is known to be the limiting precursor of CoA synthesis^24^. Furthermore, a recent study revealed that the concentration of pantothenate in the culture medium can dramatically affect the production rate of farnesene, a sesquiterpene isoprenoid polymer of acetyl-CoA, which is synthesized via the mevalonate pathway^25^. Importantly, this effect is mediated primarily at the metabolic level and not the transcriptional level in the *S. cerevisiae* strain that was investigated, which had been genetically engineered to generate high levels of farnesene^24^. MF is also a member of the sesquiterpenoid family of compounds and is synthesized initially from acetyl-CoA via the mevalonate synthesis pathway. Moreover, our previous transcriptome analysis revealed that expression levels of genes involved in the mevalonate pathway and farnesoid metabolism pathway were not changed in response to the short-day condition^11^, which is consistent with the observations in *S. cerevisiae*^24^. Although further investigations are necessary to elucidate the causal relationship between accumulation of pantothenate and the MF biosynthesis pathways in male offspring production in the *D. pulex* WTN6 strain, mechanisms underlying the activation of the farnesoid biosynthesis pathway in response to pantothenate might be widely conserved among organisms.

We conducted a metabolome analysis to shed light on the molecular components underlying the ESD system in *D. pulex*. We putatively annotated several candidate metabolites and their potential pathways involved in the ESD of *D. pulex*, including pantothenate as a potential source of MF biosynthesis, and glutamate, the ligand for the ionotropic glutamate receptor whose transcription has previously been shown to alter in response to a shortened photoperiod^11^. Moreover, the glycogen synthesis pathway might be implicated in the downstream pathway of MF signaling. Although further investigations into the molecular function of the pantothenate and glycogen synthesis pathways will be required, the combination of metabolic and transcriptional profiles enabled a deeper understanding of how external environmental stimuli affects physiological changes in mother daphnids triggered for sexual fate determination of their offspring. Our findings not only provide important molecular clues involved in the signaling cascades regulating male offspring production in response to the short-day condition in *D. pulex*, but also contribute to elucidating how animals transmit information from the external changeable environment and transform it into phenotypic alterations.

## Materials and Methods

### Male- and female-inducing conditions in *Daphnia pulex* WTN6 strain

The *D. pulex* WTN6 strain was collected at West Trenton (MO, USA) in 2006, and obtained from the Center for Genomics and Bioinformatics (Indiana University, USA). Animals were maintained in dechlorinated freshwater, which was aerated and filtered through activated carbon for two weeks, at 18 °C. The water hardness and dissolved oxygen concentration were 60 mg/L (as CaCO_3_) and 91.5% as measured by chelatometric titration (Kyoritsu Chemical-check Lab., Corp., Tokyo, Japan) and a dissolved oxygen meter (SevenGo pro SG9, Mettler-Toledo, LLC., Ohio, USA), respectively. A 0.04-mL suspension of 4.3 × 10^8^ cells mL^−1^ *Chlorella vulgaris* was added daily to each culture of 40 individual daphnids in 2L beakers as a food source. The WTN6 strain was selected for this study as it is possible to induce the production of female or male offspring by rearing under 14 h light : 10 h dark or 10 h light : 14 h dark conditions, respectively^10^,^14^.

### Morphometric analysis based on body length

One-month-old *D. pulex* reared under long-day (n = 10) and short-day (n = 11) conditions were each held between a glass slide and glass cover slip, and images were captured using a light microscope (CKX41, Olympus, Tokyo, Japan), CCD camera (DP-72, Olympus) and analysis software (CellSens Standard, Olympus). Body lengths were measured from the top of the head to the proximal region of the tail-spine using ImageJ software^26^. Measured body-lengths of adults raised under each condition were assessed by Student’s *t*-test.

### Sampling and extraction of polar metabolites

Each individual daphnid was cultured in 50 mL of freshwater under the long-day or short-day conditions, and then flash-frozen in liquid nitrogen during the critical period for sex determination (50 hours after ovulation) at one month of age (*i.e.*, after ovulating at least 8 times). Three individual animals were combined to form sufficient biomass for one biological replicate, and ten experiments were conducted independently yielding ten biological replicates for each of the two conditions. Polar metabolites were extracted from *D. pulex* samples using a two-step, biphasic method as detailed previously^27^. Briefly, each frozen sample was homogenized using a Precellys-24 ceramic bead-based homogenizer (Stretton Scientific Ltd., UK) in 448 μL of a 5:2 mixture of methanol:water (both high-performance liquid chromatography [HPLC] grade). Homogenate was then transferred into a 1.8 mL glass vial and further volumes of water (160 μL) and chloroform (320 μL) were added to yield a final solvent ratio of 2:2:1.8 methanol:chloroform:water^27^. This mixture was vortex-mixed and centrifuged (4000-*g* rcf, 10 min) yielding a biphasic separation. The upper, polar layer of each sample was split evenly (approximately 180 μL per aliquot) into two 1.5 mL Eppendorf tubes for separate mass spectrometric analyses in positive and negative ion modes. The lower, non-polar layer was not used in this study. Polar extracts were dried using a centrifugal concentrator (Thermo Savant, Holbrook, NY, USA) and stored at −80 °C prior to DIMS analysis. Two “negative controls” were created using the above homogenization and extraction protocols in the absence of biological material.

### Mass spectrometry metabolomics and spectral processing

Immediately prior to analysis, polar extracts were removed from storage at −80 °C and resuspended for Fourier transform ion cyclotron resonance (FT-ICR) DIMS analysis. One extract per sample was resuspended in 60 μL 4:1 methanol:water with 20 mM ammonium acetate for negative ion analysis, while the other was resuspended in 60 μL 4:1 methanol:water containing 0.25% formic acid for positive ion analysis. Resuspended samples were centrifuged (22,000-*g* rcf, 10 min, 4 °C) to remove particulate matter. The supernatant of each sample was then loaded as four adjacent technical replicates (5 μL per well) onto a 384 well plate (Agilent Technologies, Santa Clara, CA, USA) in a random order. From the remaining supernatant of each sample, 15 μL were removed and pooled to generate a quality control sample (QC).

All *D. pulex* samples were analyzed in both ion modes using an LTQ FT Ultra FT-ICR mass spectrometer (Thermo Fisher Scientific, Bremen, Germany) with a direct infusion, chip-based nanoelectrospray ionization source (Triversa, Advion Biosciences, Ithaca, NY, USA). Data were acquired from *m/z* 70–590 using the selected ion monitoring (SIM) stitching methodology^28^ in seven SIM windows of 100 *m/z* width and 30 *m/z* overlap. Samples were analyzed as quadruplicate technical replicates with QC samples spaced evenly throughout the analysis to allow any drift in the DIMS signal intensities to be distinguished from genuine biological differences. Once acquired, data from the three most consistent technical replicates (for each sample) were processed using the SIM stitch algorithm, as reported previously^29^,^30^. The resulting filtered matrices (one per ion mode) were probabilistic quotient normalized (PQN)^31^ and missing values were imputed using the k-nearest neighbor (KNN) algorithm^32^. The resulting matrices were analysed using univariate statistics (see below), and were transformed using the generalized logarithm^33^ prior to multivariate statistical analysis. All spectral processing was conducted using in-house scripts in Matlab (version 7, The MathWorks, Natick, MA, USA). Metabolomics data have been deposited into the MetaboLights repository (accession number: MTBLS259, accessed via link: http://www.ebi.ac.uk/metabolights/MTBLS259).

### Statistical analysis of *m/z* peak intensity matrices

Principal components analysis (PCA) was used to investigate the similarities and differences between the metabolic profiles of all the samples in an unbiased manner using the PLS-Toolbox (version 6.0.1, Eigenvector Research, Manson, WA, USA) within Matlab. Differences between the PC scores values of the two treatment groups (across the first four PCs) were assessed by a *t*-test using an in-house script in Matlab. Changes in the intensities of individual *m/z* peaks were also assessed using *t*-tests. All univariate statistical analyses were corrected using a false discovery rate (FDR) of 5% to account for multiple-testing and the results presented as adjusted p-values^17^. QC samples were excluded from these statistical analyses.

### Putative metabolite annotation and pathway analysis

Putative empirical formula(e) and KEGG (Kyoto Encyclopedia of Genes and Genomes) compound names were assigned, where possible, to the significantly changing *m/z* peaks using the transformation mapping algorithm in the Metabolite Identification Package (MI-Pack)^15^. Briefly, MI-Pack calculates accurate *m/z* values for common negative ions ([M-H]^−^, [M + ^35^Cl]^−^, [M + ^37^Cl]^−^, [M + Acetate(Ac)]^−^, [M + K-2H]^−^ and [M + Na-2H]^−^) and positive ions ([M + H]^+^, [M + Na]^+^, [M + ^39^K]^+^, [M + ^41^K]^+^) of all compounds within the KEGG compounds database. It then attempts to match these *m/z* values with the accurate masses measured in the mass spectra, within a mass error window of 1 ppm. The transformation mapping algorithm^15^ was used to increase confidence in the assigned putative metabolite names. The resulting list of significantly changing KEGG compound IDs was input into MetaboAnalyst^16^ and Fisher’s exact test was used for over-representation analysis against a background list of all putatively annotated KEGG compound IDs^34^.

### Pharmacological manipulation of *Daphnia pulex*

As described above, we discovered that pantothenate (vitamin B5) was significantly accumulated in response to the short-day condition, and hence direct exposure of daphnids to this metabolite was investigated. Calcium (+)-pantothenate (≥98%; Wako, Osaka, Japan) was dissolved in water and administrated to each 50 mL beaker of freshwater containing one adult female (at least one month old). The final concentrations of pantothenate were 20, 40, 100, 200 and 400 μM (n = 13–15 replicates per exposure concentration). Pantothenate treatment experiments were carried out using one-month age mother reared under both long-day and short-day conditions. A 20-μL suspension of 4.3 × 10^8^ cells mL^−1^ of *C. vulgaris* was added to this treatment water, and both the treatment water and algal food were renewed daily for 7 days. Differences in the percentage of mothers that produced male offspring, between control and pantothenate treated groups, were statistically analyzed by Fisher’s exact probability test with Holm’s correction, using R version 2.15.3^35^.

## Additional Information

**How to cite this article**: Toyota, K. *et al*. Metabolomics reveals an involvement of pantothenate for male production responding to the short-day stimulus in the water flea, *Daphnia pulex. Sci. Rep.* **6**, 25125; doi: 10.1038/srep25125 (2016).

## Supplementary Material

Supplementary Information

## Figures and Tables

**Table 1:** Selected list of putatively annotated peaks that change significantly in response to the short-day conditions, ranked according to their fold change.

Observed	Statistics		Annotation				
*m/z*	Adjusted p-value	Fold Change^1^	Empirical formula	Ion form	Neutral mass (Da)	Mass error	Putative metabolite name(s)
256.05916	0.0012	5.86	C9H17NO5	[M + K-2H]^−^	219.11067	−0.47	Pantothenate
			C8H15NO6	[M + Cl]^−^	221.08994	−0.71	N-acetyl-D-glucosamine, N-acetyl-D-mannosamine
			C5H12NO5P	[M + Hac-H]^−^	197.04531	−0.02	(3-(acetylhydroxyamino)propyl)phosphonic acid
260.05306	0.0012	5.52	C8H15NO6	[M + K]^+^	221.08994	−0.15	N-acetyl-D-glucosamine, N-acetyl-D-mannosamine
			C6H14NO8P	[M+H]^+^	259.04571	0.29	1-Amino-1-deoxy-scyllo-inositol 4-phosphate, Aminofructose 6-phosphate, D-glucosamine 6-phosphate, Kanosamine 6-phosphate, alpha-D-glucosamine 1-phosphate
156.04209	0.0053	5.40	C3H10NO4P	[M+H]^+^	155.03475	0.43	D-1-aminopropan-2-ol O-phosphate, N-methylethanolamine phosphate
			C5H11NO2	[M+K]^+^	117.07898	−0.31	4-Methylaminobutyrate, 5-Aminopentanoate, Betaine, L-Valine
			C8H7NO	[M+Na]^+^	133.05276	0.67	4-Hydroxyphenylacetonitrile, Indolin-2-one
258.05621	0.0133	4.29	C8H15NO6	[M+(^37^Cl)]^−^	221.08994	−0.70	N-acetyl-D-glucosamine, N-acetyl-D-mannosamine

Full list is presented in Table S1.

^1^Fold change in intensity from long-day to short-day exposure groups.
