# Supplementary material for: Metabolomics reveals an involvement of pantothenate for male production responding to the short-day stimulus in the water flea, Daphnia pulex
Source: Sci Rep. 2016 Apr 26;6:25125. doi: 10.1038/srep25125 (PMC4844948; doi:10.1038/srep25125)
Supplement: Supplementary Information [file srep25125-s1.doc]

**Metabolomics reveals an involvement of pantothenate for male production responding to the short-day stimulus in the water flea, *Daphnia pulex***

Kenji Toyota^1,2,§^, Alex Gavin^3,§^, Shinichi Miyagawa^1^, Mark R. Viant^3^, Taisen Iguchi^1,*^

^1^Okazaki Institute for Integrative Bioscience, National Institute for Basic Biology, National Institutes of Natural Sciences, and Department of Basic Biology, Faculty of Life Science, SOKENDAI (The Graduate University for Advanced Studies), 5-1 Higashiyama, Myodaiji, Okazaki, Aichi 444-8787, Japan

^2^Environmental Genomics Group. School of Biosciences, University of Birmingham, Edgbaston, Birmingham B15 2TT, U.K.

^3^Environmental Metabolomics Research Group, School of Biosciences, University of Birmingham, Edgbaston, Birmingham B15 2TT, U.K.

^§^These authors contributed equally.

^*^Corresponding author


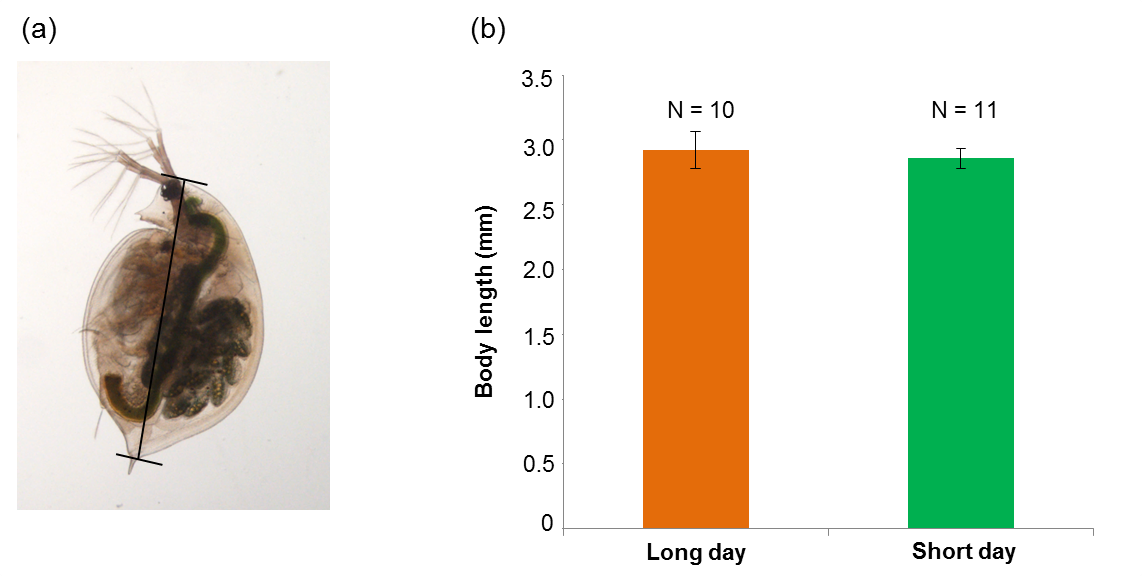


**Supplemental Figure S1**

Solid line shows the body length measurement used for the morphometric comparison of culture conditions (**a**). Body length measurements of *D. pulex* (one month old) reared under the long-day and short-day conditions (**b**). Bars indicate one standard deviation. Numbers indicate the biological replicates.

**Supplemental Table S1. List of all significantly up-regulated and putatively annotated peaks in response to the short-day condition.**

| **Observed** | **Statistics** | | **Identification** | | | | |
| --- | --- | --- | --- | --- | --- | --- | --- |
| **m/z** | **Adjusted p-values** | **Fold Change^1^** | **Empirical formula** | **Ion form** | **Neutral mass (Da)** | **Mass error** | **Putative metabolite name(s)** |
| 628.0568 | 0.0011 | 1.32 | C17H27N3O17P2 | [M+Na-2H]- | 607.0816 | 0.85 | UDP-N-acetyl-D-galactosamine, UDP-N-acetyl-D-glucosamine, UDP-N-acetyl-D-mannosamine |
| 606.0746 | < 0.001 | 1.27 | C17H27N3O17P2 | [M-H]- | 607.0816 | 0.54 |
| 199.9692 | 0.0457 | 1.56 | C3H7NO5S2 | [M-H]- | 200.9766 | -0.26 | S-Sulfo-L-cysteine |
| 245.0431 | 0.0011 | 1.75 | C11H12O5 | [M+Na-2H]- | 224.0685 | -0.34 | Sinapate |
| 383.1144 | 0.0031 | 1.43 | C14H20N6O5S | [M-H]- | 384.1216 | 0.22 | S-Adenosyl-L-homocysteine |
| 385.1289 | 0.0241 | 1.27 | C14H20N6O5S | [M+H]+ | 384.1216 | 0.19 |
| 375.1309 | 0.0316 | 1.33 | C17H20N4O6 | [M-H]- | 376.1383 | -0.21 | Riboflavin |
| 127.0512 | < 0.001 | 1.19 | C3H4N2 | [M+Hac-H]- | 68.0374 | -0.72 | Pyrazole |
| 118.9307 | 0.0363 | 1.53 | H3O3P | [M+K-2H]- | 81.982 | 0.59 | Phosphonate |
| 206.0167 | 0.0019 | 2.61 | C4H12NO4P | [M+(37Cl)]- | 169.0504 | -0.77 | Phosphodimethylethanolamine |
| 204.0198 | < 0.001 | 1.94 | C4H12NO4P | [M+Cl]- | 169.0504 | 0.2 |
| 192.0397 | 0.0447 | 1.65 | C4H12NO4P | [M+Na]+ | 169.0504 | 0.37 |
| 256.0592 | 0.0012 | 5.86 | C9H17NO5 | [M+K-2H]- | 219.1107 | -0.47 | Pantothenate |
| 132.9463 | 0.0255 | 1.55 | H3O4P | [M+Hac-H]- | 97.9769 | -0.33 | Orthophosphate |
| 156.9907 | 0.029 | 1.36 | H3O4P | [M+Hac-H]- | 97.9769 | -0.33 |
| 134.9434 | 0.0312 | 1.49 | H3O4P | [M+(37Cl)]- | 97.9769 | 0.08 |
| 429.1187 | 0.008 | 1.43 | C22H22O9 | [M-H]- | 430.1264 | -1 | Ononin |
| 278.0434 | < 0.001 | 2.02 | C14H11NO4 | [M+Na-2H]- | 257.0688 | -0.17 | N-Benzoyl-4-hydroxyanthranilate |
| 200.0483 | 0.0084 | 1.43 | C10H13NO | [M+K-2H]- | 163.0997 | 0 | N-Acetylphenylethylamine |
| 256.0592 | 0.0012 | 5.86 | C8H15NO6 | [M+Cl]- | 221.0899 | -0.71 | N-Acetyl-D-glucosamine, N-Acetyl-D-mannosamine |
| 260.0531 | 0.0012 | 5.52 | C8H15NO6 | [M+K]+ | 221.0899 | -0.15 |
| 258.0562 | 0.0133 | 4.29 | C8H15NO6 | [M+(37Cl)]- | 221.0899 | -0.7 |
| 244.0791 | 0.013 | 2.12 | C8H15NO6 | [M+Na]+ | 221.0899 | -0.21 |
| 188.1757 | 0.0211 | 1.63 | C9H21N3O | [M+H]+ | 187.1685 | -0.2 | N1-Acetylspermidine, N8-Acetylspermidine |
| 380.052 | 0.0386 | 1.82 | C11H22NO7PS | [M+(37Cl)]- | 343.0855 | 0.25 | N-(7-Mercaptoheptanoyl)threonine 3-O-phosphate |
| 378.055 | 0.0069 | 1.34 | C11H22NO7PS | [M+Cl]- | 343.0855 | 0.3 |
| 262.1198 | < 0.0001 | 3.85 | C11H13N3O | [M+Hac-H]- | 203.1059 | 0.17 | L-Tryptophanamide |
| 225.0644 | 0.0349 | 1.39 | C11H12N2O2 | [M+Na-2H]- | 204.0899 | -0.87 | L-Tryptophan, Tryptophan |
| 203.0825 | 0.0014 | 1.26 | C11H12N2O2 | [M-H]- | 204.0899 | -0.35 |
| 166.0532 | 0.0011 | 2.86 | C5H11NO3S | [M+H]+ | 165.046 | -0.26 | L-Methionine (R)-S-oxide, L-Methionine (S)-S-oxide, L-Methionine S-oxide |
| 202.0122 | 0.0055 | 1.27 | C5H11NO3S | [M+(37Cl)]- | 165.046 | -0.88 |
| 248.0539 | 0.0016 | 1.53 | C10H13NO5 | [M+Na-2H]- | 227.0794 | -0.7 | L-Arogenate |
| 226.0719 | 0.0075 | 1.50 | C10H13NO5 | [M-H]- | 227.0794 | -0.79 | L-Arogenate |
| 267.0733 | 0.0139 | 1.42 | C10H12N4O5 | [M-H]- | 268.0808 | -0.69 | Inosine |
| 258.0382 | < 0.001 | 1.46 | C4H10NO6P | [M+Hac-H]- | 199.0246 | -0.74 | Iminoerythrose 4-phosphate, L-Threonine O-3-phosphate, O-Phospho-L-homoserine, O-Phosphorylhomoserine |
| 282.0842 | < 0.001 | 1.68 | C10H13N5O5 | [M-H]- | 283.0917 | -0.62 | Guanosine |
| 320.0582 | 0.0305 | 1.66 | C10H13N5O5 | [M+(37Cl)]- | 283.0917 | 0.12 |
| 318.0612 | 0.0183 | 1.60 | C10H13N5O5 | [M+Cl]- | 283.0917 | 0.28 |
| 342.1056 | 0.0018 | 1.53 | C10H13N5O5 | [M+Hac-H]- | 283.0917 | 0.08 |
| 604.0702 | 0.0017 | 1.46 | C16H25N5O16P2 | [M-H]- | 605.0772 | 0.47 | GDP-L-galactose, GDP-L-gulose, GDP-glucose, GDP-mannose |
| 256.0592 | 0.0012 | 5.86 | C5H12NO5P | [M+Hac-H]- | 197.0453 | -0.02 | FR 900098 |
| 173.0567 | 0.003 | 1.59 | C6H10N2O4 | [M-H]- | 174.0641 | -0.59 | Formylisoglutamine, N-Formimino-L-glutamate |
| 175.0713 | 0.0424 | 1.56 | C6H10N2O4 | [M+H]+ | 174.0641 | -0.25 |
| 207.0775 | 0.0228 | 1.40 | C10H12N2O3 | [M-H]- | 208.0848 | -0.18 | Formyl-5-hydroxykynurenamine, L-Kynurenine |
| 267.0984 | 0.0066 | 1.37 | C10H12N2O3 | [M+Hac-H]- | 208.0848 | -0.77 |
| 221.9754 | 0.0247 | 1.64 | C3H8NO6P | [M+(37Cl)]- | 185.0089 | 0.14 | D-O-Phosphoserine, O-Phospho-L-serine |
| 219.9784 | 0.003 | 1.62 | C3H8NO6P | [M+Cl]- | 185.0089 | 0.32 |
| 150.0583 | 0.0145 | 1.20 | C5H11NO2S | [M+H]+ | 149.0511 | -0.18 | D-Methionine, L-Methionine |
| 394.0771 | 0.0155 | 1.88 | C9H14N5O7P | [M+Hac-H]- | 335.0631 | 0.28 | Dihydroneopterin phosphate |
| 448.0008 | 0.0013 | 1.53 | C10H15N5O9P2 | [M+(37Cl)]- | 411.0345 | -0.44 | dADP |
| 226.0719 | 0.0075 | 1.50 | C8H9NO3 | [M+Hac-H]- | 167.0582 | -0.79 | D-4-Hydroxyphenylglycine, Isopyridoxal, L-4-Hydroxyphenylglycine, Pyridoxal |
| 156.0421 | 0.0053 | 5.40 | C3H10NO4P | [M+H]+ | 155.0347 | 0.43 | D-1-Aminopropan-2-ol O-phosphate, N-Methylethanolamine phosphate |
| 221.0601 | 0.005 | 1.71 | C7H14N2O4S | [M-H]- | 222.0674 | -0.15 | Cystathionine, L-Cystathionine |
| 445.0533 | 0.0068 | 1.23 | C11H20N4O11P2 | [M-H]- | 446.0604 | 0.45 | CDP-ethanolamine |
| 261.0758 | < 0.001 | 1.53 | C9H14N4O3 | [M+Cl]- | 226.1066 | -0.74 | Carnosine |
| 221.0853 | < 0.0001 | 2.43 | C12H14N2 | [M+Cl]- | 186.1157 | 0.73 | Calligonine |
| 209.081 | 0.0012 | 1.49 | C6H14N4O2 | [M+Cl]- | 174.1117 | -0.32 | Amino acid(Arg-), D-Arginine, L-Arginine |
| 211.0781 | 0.0013 | 1.48 | C6H14N4O2 | [M+(37Cl)]- | 174.1117 | -0.23 |
| 261.0879 | 0.0119 | 1.33 | C11H10N2O2 | [M+Hac-H]- | 202.0742 | -0.66 | alpha,beta-Didehydrotryptophan |
| 266.0893 | 0.0011 | 1.50 | C10H13N5O4 | [M-H]- | 267.0968 | -0.71 | Adenosine, Deoxyguanosine |
| 447.1633 | 0.0041 | 1.41 | C21H30O9 | [M+Na-2H]- | 426.189 | -0.72 | Abscisic acid glucose ester |
| 221.0853 | < 0.0001 | 2.43 | C7H14O2S | [M+Hac-H]- | 162.0715 | -0.21 | 7-Mercaptoheptanoic acid |
| 247.1662 | 0.0063 | 1.65 | C9H20N2O2 | [M+Hac-H]- | 188.1525 | -0.65 | 7,8-Diaminononanoate |
| 220.1012 | < 0.0001 | 3.16 | C7H15NOS | [M+Hac-H]- | 161.0874 | -0.36 | 6-Methylthiohexanaldoxime |
| 176.0927 | < 0.001 | 1.64 | C5H11NO2 | [M+Hac-H]- | 117.079 | -0.64 | 5-Aminopentanoate, Betaine, L-Norvaline, L-Valine |
| 116.0716 | < 0.001 | 1.41 | C5H11NO2 | [M-H]- | 117.079 | -0.88 |
| 212.033 | 0.0018 | 1.32 | C10H9NO3 | [M+Na-2H]- | 191.0582 | 0.41 | 5,6-Dihydroxy-3-methyl-2-oxo-1,2-dihydroquinoline, 5-Hydroxyindoleacetate, 5-Phenyl-1,3-oxazinane-2,4-dione |
| 127.0512 | < 0.001 | 1.19 | C5H8N2O2 | [M-H]- | 128.0586 | -0.72 | 5,6-Dihydrothymine, gamma-Amino-gamma-cyanobutanoate |
| 156.0421 | 0.0053 | 5.40 | C5H11NO2 | [M+K]+ | 117.079 | -0.31 | 4-Methylaminobutyrate, 5-Aminopentanoate, Betaine, L-Valine |
| 140.0682 | < 0.001 | 2.11 | C5H11NO2 | [M+Na]+ | 117.079 | -0.15 |
| 118.0863 | < 0.001 | 1.30 | C5H11NO2 | [M+H]+ | 117.079 | -0.05 |
| 156.0421 | 0.0053 | 5.40 | C8H7NO | [M+Na]+ | 133.0528 | 0.67 | 4-Hydroxyphenylacetonitrile, Indolin-2-one |
| 212.033 | 0.0018 | 1.32 | C6H11NO5 | [M+Cl]- | 177.0637 | -0.59 | 4-Hydroxy-4-methylglutamate |
| 200.0483 | 0.0084 | 1.43 | C9H11NO2 | [M+Cl]- | 165.079 | -0.3 | 4-Hydroxy-1-(3-pyridinyl)-1-butanone, D-Phenylalanine, L-Phenylalanine |
| 202.0454 | 0.0312 | 1.38 | C9H11NO2 | [M+(37Cl)]- | 165.079 | -0.25 |
| 164.0716 | 0.002 | 1.27 | C9H11NO2 | [M-H]- | 165.079 | -0.87 |
| 138.055 | 0.0127 | 1.37 | C7H7NO2 | [M+H]+ | 137.0477 | -0.04 | 4-Aminobenzoate, Anthranilate, N-Methylnicotinate |
| 171.0166 | 0.031 | 1.68 | C4H8N2O3 | [M+K]+ | 132.0535 | -0.19 | 3-Ureidopropionate, L-Asparagine, N-Carbamoylsarcosine |
| 131.0461 | < 0.001 | 1.47 | C4H8N2O3 | [M-H]- | 132.0535 | -0.66 | 3-Ureidopropionate, Glycylglycine, L-Asparagine, N-Carbamoylsarcosine |
| 145.0618 | 0.0275 | 1.25 | C5H10N2O3 | [M-H]- | 146.0691 | -0.67 | 3-Ureidoisobutyrate, D-Glutamine, Isoglutamine, L-Glutamine |
| 323.0286 | 0.0174 | 1.24 | C9H13N2O9P | [M-H]- | 324.0359 | -0.14 | 3-UMP, Pseudouridine 5-phosphate, UMP |
| 147.0451 | 0.0273 | 1.32 | C9H8O2 | [M-H]- | 148.0524 | -0.7 | 3-Hydroxy-1-indanone, 3-Isochromanone, 4-Hydroxycinnamyl aldehyde, Dihydrocoumarin, trans-Cinnamate |
| 206.0458 | 0.006 | 1.38 | C10H9NO4 | [M-H]- | 207.0532 | -0.3 | 3-Amino-4,7-dihydroxy-8-methylcoumarin, 4-(2-Aminophenyl)-2,4-dioxobutanoate |
| 216.0432 | 0.0303 | 1.53 | C9H11NO3 | [M+Cl]- | 181.0739 | -0.26 | 3-Amino-3-(4-hydroxyphenyl)propanoate, L-Tyrosine |
| 180.0665 | < 0.001 | 1.40 | C9H11NO3 | [M-H]- | 181.0739 | -0.65 |
| 202.0485 | 0.0055 | 1.29 | C9H11NO3 | [M+Na-2H]- | 181.0739 | -0.21 |
| 190.0388 | 0.0366 | 1.39 | C6H9N3O2 | [M+Cl]- | 155.0695 | -0.31 | 3-(Pyrazol-1-yl)-L-alanine, L-Histidine |
| 192.0359 | 0.0319 | 1.31 | C6H9N3O2 | [M+(37Cl)]- | 155.0695 | -0.25 |
| 277.1113 | 0.0195 | 1.31 | C10H18O3S | [M+Hac-H]- | 218.0977 | -0.72 | 2-Oxo-9-methylthiononanoic acid |
| 168.0277 | 0.0335 | 1.28 | C5H9NO4 | [M+Na-2H]- | 147.0532 | -0.64 | 2-Oxo-4-hydroxy-5-aminovalerate, D-Glutamate, DL-Glutamate, L-4-Hydroxyglutamate semialdehyde, L-Glutamate, L-threo-3-Methylaspartate, N-(Carboxymethyl)-D-alanine, O-Acetyl-L-serine |
| 202.0122 | 0.0055 | 1.27 | C8H7NO4 | [M+Na-2H]- | 181.0375 | 0.31 | 2-Methyl-3-hydroxy-5-formylpyridine-4-carboxylate, DIBOA |
| 163.0399 | < 0.0001 | 1.39 | C9H8O3 | [M-H]- | 164.0473 | -0.79 | 2-Hydroxy-3-phenylpropenoate, 3-Coumaric acid, 4-Coumarate, Benzoyl acetate, Caffeic aldehyde, Phenylpyruvate, trans-2-Hydroxycinnamate |
| 184.059 | 0.0076 | 1.33 | C6H13NO4 | [M+Na-2H]- | 163.0845 | -0.48 | 2-Deoxy-scyllo-inosamine |
| 216.0279 | 0.0193 | 1.22 | C9H9NO4 | [M+Na-2H]- | 195.0532 | 0.34 | 2-Carboxy-2,3-dihydro-5,6-dihydroxyindole, Dopaquinone |
| 211.0724 | 0.0248 | 1.19 | C7H8N2O2 | [M+Hac-H]- | 152.0586 | -0.2 | 2-Amino-4-nitrotoluene, 4-Amino-2-nitrotoluene |
| 173.0567 | 0.003 | 1.59 | C4H6N2O2 | [M+Hac-H]- | 114.0429 | -0.59 | 2,5-Dioxopiperazine, 3-Cyano-L-alanine, 5,6-Dihydrouracil, N-Methylhydantoin |
| 333.0593 | < 0.0001 | 2.36 | C9H19O11P | [M-H]- | 334.0665 | 0.07 | 2-(alpha-D-Galactosyl)-sn-glycerol 3-phosphate, 2-(beta-D-Glucosyl)-sn-glycerol 3-phosphate, "alpha-D-Galactosyl-(1,1)-sn-glycerol 3-phosphate", sn-glycero-3-Phospho-1-inositol |
| 277.1113 | 0.0195 | 1.31 | C12H22O5S | [M-H]- | 278.1188 | -0.72 | 2-(7-Methylthio)heptylmalic acid, 3-(7-Methylthio)heptylmalic acid |
| 318.0612 | 0.0183 | 1.60 | C11H15N5O4 | [M+K-2H]- | 281.1124 | 0.47 | 1-Methyladenosine |
| 333.0593 | < 0.0001 | 2.36 | C7H15O9P | [M+Hac-H]- | 274.0454 | 0.07 | 1-Deoxy-D-altro-heptulose 7-phosphate |
| 216.0279 | 0.0193 | 1.22 | C6H13NO5 | [M+K-2H]- | 179.0794 | -0.37 | 1-Amino-1-deoxy-scyllo-inositol, D-Galactosamine, D-Glucosamine, Kanosamine |
| 260.0531 | 0.0012 | 5.52 | C6H14NO8P | [M+H]+ | 259.0457 | 0.29 | 1-Amino-1-deoxy-scyllo-inositol 4-phosphate, Aminofructose 6-phosphate, D-Glucosamine 6-phosphate, Kanosamine 6-phosphate, alpha-D-Glucosamine 1-phosphate |
| 258.0382 | < 0.001 | 1.46 | C6H14NO8P | [M-H]- | 259.0457 | -0.74 | 1-Amino-1-deoxy-scyllo-inositol 4-phosphate, Aminofructose 6-phosphate, D-Galactosamine 6-phosphate, D-Glucosamine 6-phosphate, Kanosamine 6-phosphate, alpha-D-Glucosamine 1-phosphate |
| 210.0771 | 0.0422 | 1.18 | C8H9NO2 | [M+Hac-H]- | 151.0633 | -0.35 | (Z)-4-Hydroxyphenylacetaldehyde-oxime, 2-Descarboxy-cyclo-dopa, Dopamine quinone, N-Methylanthranilate |
| 185.0686 | 0.0231 | 1.67 | C6H14N2O2 | [M+K]+ | 146.1055 | -0.26 | (3S)-3,6-Diaminohexanoate, (3S,5S)-3,5-Diaminohexanoate, 2,5-Diaminohexanoate, D-Lysine, L-Lysine |
| 145.0982 | < 0.001 | 1.31 | C6H14N2O2 | [M-H]- | 146.1055 | -0.63 |
| 169.0947 | 0.0312 | 1.29 | C6H14N2O2 | [M+Na]+ | 146.1055 | -0.29 |
| 147.1128 | 0.0105 | 1.18 | C6H14N2O2 | [M+H]+ | 146.1055 | -0.23 |
| 166.0639 | 0.0033 | 1.69 | C6H13NO2 | [M+Cl]- | 131.0946 | -0.61 | (3R)-beta-Leucine, 6-Aminohexanoate, L-Isoleucine, L-Leucine, L-Norleucine |
| 130.0872 | < 0.001 | 1.41 | C6H13NO2 | [M-H]- | 131.0946 | -0.87 |
| 132.1019 | 0.0232 | 1.18 | C6H13NO2 | [M+H]+ | 131.0946 | -0.12 |
| 216.0432 | 0.0303 | 1.53 | C10H13NO2 | [M+K-2H]- | 179.0946 | 0.02 | (-)-Salsolinol, L-Homophenylalanine |

**Supplemental Table S2**. List of all significantly down-regulated and putatively annotated peaks in response to the short-day condition.

| **Observed** | **Statistics** | | **Identification** | | | | |
| --- | --- | --- | --- | --- | --- | --- | --- |
| **m/z** | **Adjusted p-values** | **Fold Change** | **Empirical formula** | **Ion form** | **Neutral mass (Da)** | **Mass error** | **Putative metabolite name(s)** |
| 377.0799 | 0.0025 | 0.60 | C19H18O6 | [M+Cl]- | 342.1103 | 0.31 | UWM6 |
| 250.1117 | < 0.001 | 0.48 | C8H17NO2S | [M+Hac-H]- | 191.098 | -0.78 | Trihomomethionine |
| 195.0509 | 0.0135 | 0.76 | C4H8O5 | [M+Hac-H]- | 136.0372 | -0.45 | Threonate |
| 251.0955 | < 0.0001 | 0.29 | C13H16N2O | [M+Cl]- | 216.1263 | -0.54 | Tetrahydroharmine |
| 251.0771 | 0.0183 | 0.72 | C7H12O6 | [M+Hac-H]- | 192.0634 | -0.53 | Quinate |
| 215.0327 | 0.0246 | 0.72 | C3H9O5P | [M+Hac-H]- | 156.0188 | 0.29 | Propane-1,2-diol 1-phosphate |
| 228.0642 | 0.0015 | 0.80 | C4H12NO4P | [M+Hac-H]- | 169.0504 | -0.18 | Phosphodimethylethanolamine |
| 220.0815 | 0.0012 | 0.46 | C8H13NO6 | [M+H]+ | 219.0743 | -0.25 | O-Succinyl-L-homoserine |
| 218.067 | < 0.001 | 0.55 | C8H13NO6 | [M-H]- | 219.0743 | -0.24 |
| 215.0308 | 0.0386 | 0.72 | C5H4N2O4 | [M+Hac-H]- | 156.0171 | -0.94 | Orotate, Uracil 5-carboxylate |
| 276.119 | 0.0061 | 0.78 | C10H17N3O6 | [M+H]+ | 275.1117 | -0.16 | N-Succinyl-L-citrulline |
| 174.076 | < 0.001 | 0.45 | C7H11NO4 | [M+H]+ | 173.0688 | -0.26 | N-Acetyl-L-glutamate 5-semialdehyde |
| 172.0614 | 0.0026 | 0.61 | C7H11NO4 | [M-H]- | 173.0688 | -0.71 |
| 216.0361 | 0.0517 | 0.74 | C8H9N3O2 | [M+(37Cl)]- | 179.0695 | 0.79 | N-Acetylisoniazid |
| 228.0642 | 0.0015 | 0.80 | C11H13NO3 | [M+Na-2H]- | 207.0895 | -0.01 | N-Acetyl-D-phenylalanine, N-Acetyl-L-phenylalanine |
| 260.074 | < 0.001 | 0.33 | C8H15NO7 | [M+Na]+ | 237.0849 | -0.21 | N-Acetyl-D-glucosaminate |
| 276.048 | 0.0014 | 0.35 | C8H15NO7 | [M+K]+ | 237.0849 | -0.19 |
| 238.0921 | < 0.001 | 0.49 | C8H15NO7 | [M+H]+ | 237.0849 | -0.17 |
| 350.1126 | 0.0186 | 0.67 | C12H22N3O7P | [M-H]- | 351.1195 | 0.99 | N-Acetyldemethylphosphinothricin tripeptide |
| 377.0857 | 0.0182 | 0.56 | C13H24O10 | [M+K-2H]- | 340.137 | 0.27 | Methyl-2-alpha-L-fucopyranosyl-beta-D-galactoside |
| 136.0427 | < 0.001 | 0.48 | C4H9NO2S | [M+H]+ | 135.0354 | -0.13 | L-Homocysteine |
| 249.08 | < 0.0001 | 0.34 | C13H14N2O | [M+Cl]- | 214.1106 | 0.02 | Harmaline |
| 251.0771 | 0.0183 | 0.72 | C13H14N2O | [M+(37Cl)]- | 214.1106 | 0.18 |
| 210.0632 | 0.0435 | 0.60 | C5H5N5O | [M+Hac-H]- | 151.0494 | -0.3 | Guanine |
| 187.0223 | 0.0092 | 0.72 | C5H10O6 | [M+Na-2H]- | 166.0477 | -0.47 | D-Ribonate |
| 208.0649 | 0.027 | 0.74 | C5H11NO2S | [M+Hac-H]- | 149.0511 | -0.17 | D-Methionine, L-Methionine |
| 203.0526 | 0.0226 | 0.68 | C6H12O6 | [M+Na]+ | 180.0634 | -0.3 | D-Fructose, D-Galactose, D-Glucose, D-Mannose, alpha-D-Glucose, beta-D-Fructose, beta-D-Glucose, myo-Inositol |
| 379.0827 | 0.0144 | 0.55 | C19H12O5 | [M+Hac-H]- | 320.0685 | 0.93 | Dehydrorabelomycin |
| 209.0666 | < 0.001 | 0.81 | C5H10O5 | [M+Hac-H]- | 150.0528 | -0.38 | D-Arabinose, D-Lyxose, D-Ribose, D-Ribulose, D-Xylose, D-Xylulose, L-Arabinose, L-Lyxose, L-Ribulose, L-Xylulose, beta-D-Ribofuranose, beta-D-Ribopyranose |
| 255.072 | 0.0013 | 0.65 | C6H12O7 | [M+Hac-H]- | 196.0583 | -0.74 | D-Altronate, D-Gluconic acid, D-Mannonate |
| 195.0509 | 0.0135 | 0.76 | C6H12O7 | [M-H]- | 196.0583 | -0.45 |
| 689.2117 | 0.0011 | 0.33 | C24H42O21 | [M+Na]+ | 666.2219 | 0.82 | Cellotetraose, Isolychnose, Lychnose, Stachyose |
| 705.1855 | < 0.001 | 0.37 | C24H42O21 | [M+Na]+ | 666.2219 | 0.82 |
| 703.189 | 0.0239 | 0.48 | C24H42O21 | [M+(37Cl)]- | 666.2219 | 0.93 |
| 325.113 | < 0.001 | 0.45 | C12H20O10 | [M+H]+ | 324.1057 | 0.07 | Bis-D-fructose 2,1:2,1-dianhydride |
| 323.0985 | 0.002 | 0.53 | C12H20O10 | [M-H]- | 324.1057 | 0.36 |
| 443.1408 | < 0.001 | 0.43 | C14H24O12 | [M+Hac-H]- | 384.1268 | 0.29 | Acetyl-maltose |
| 255.072 | 0.0013 | 0.65 | C12H14N2O2 | [M+(37Cl)]- | 218.1055 | -0.04 | Abrine, L-2-Methyltryptophan, N-Acetylserotonin |
| 481.0968 | 0.0016 | 0.53 | C12H23O14P | [M+Hac-H]- | 422.0825 | 0.85 | 6-Phospho-beta-D-glucosyl-(1,4)-D-glucose, Lactose 6-phosphate, Maltose 6-phosphate, Sucrose 6-phosphate, alpha,alpha-Trehalose 6-phosphate, beta-D-Fructofuranosyl-alpha-D-mannopyranoside 6F-phosphate |
| 230.0425 | 0.0011 | 0.33 | C5H12NO7P | [M+H]+ | 229.0351 | 0.27 | 5-Phosphoribosylamine |
| 226.0486 | < 0.0001 | 0.33 | C11H11NO3 | [M+Na-2H]- | 205.0739 | 0.17 | 5-Methoxyindoleacetate, Indolelactate |
| 215.0327 | 0.0246 | 0.72 | C10H10O4 | [M+Na-2H]- | 194.0579 | 0.47 | 5-Hydroxyconiferaldehyde, 6-Hydroxymellein, Ferulate |
| 218.067 | < 0.001 | 0.55 | C6H9NO4 | [M+Hac-H]- | 159.0532 | -0.24 | 4-Methylene-L-glutamate |
| 249.08 | < 0.0001 | 0.34 | C8H14O3S | [M+Hac-H]- | 190.0664 | -0.81 | 2-Oxo-7-methylthioheptanoic acid |
| 230.0425 | 0.0011 | 0.33 | C10H9NO4 | [M+Na]+ | 207.0532 | 0.43 | 2-Formaminobenzoylacetate, 4-(2-Aminophenyl)-2,4-dioxobutanoate |
| 179.056 | < 0.0001 | 0.48 | C6H12O6 | [M-H]- | 180.0634 | -0.52 | 2-Deoxy-D-gluconate, D-Aldose, D-Fructose, D-Fuconate, D-Galactose, D-Glucose, D-Hexose, D-Mannose, D-Sorbose, D-Tagatose, L-Fuconate, L-Galactose, L-Rhamnonate, L-Sorbose, alpha-D-Glucose, beta-D-Fructose, beta-D-Glucose, myo-Inositol, scyllo-Inositol |
| 239.0771 | < 0.0001 | 0.56 | C6H12O6 | [M+Hac-H]- | 180.0634 | -0.73 |
| 217.0298 | 0.0235 | 0.71 | C6H12O6 | [M+(37Cl)]- | 180.0634 | -0.42 |
| 215.0327 | 0.0246 | 0.72 | C6H12O6 | [M+Cl]- | 180.0634 | -0.52 |
| 215.0327 | 0.0246 | 0.72 | C5H13O7P | [M-H]- | 216.0399 | 0.29 | 2-C-Methyl-D-erythritol 4-phosphate |
| 190.072 | < 0.001 | 0.29 | C7H13NO5 | [M-H]- | 191.0794 | -0.57 | 2-Amino-3,7-dideoxy-D-threo-hept-6-ulosonic acid |
| 228.0457 | < 0.0001 | 0.31 | C7H13NO5 | [M+(37Cl)]- | 191.0794 | -0.77 |
| 226.0486 | < 0.0001 | 0.33 | C7H13NO5 | [M+Cl]- | 191.0794 | -0.78 |
| 212.054 | 0.0214 | 0.82 | C7H13NO5 | [M+Na-2H]- | 191.0794 | -0.44 |
| 365.1055 | < 0.001 | 0.45 | C12H22O11 | [M+Na]+ | 342.1162 | 0.23 | 2-alpha-D-Glucosyl-D-glucose, Cellobiose, D-Glucosyl-D-mannose, Epimelibiose, Inulobiose, Isomaltose, Lactose, Laminaribiose, Maltose, Melibiose, Nigerose, Palatinose, Sucrose, alpha,alpha-Trehalose, alpha-D-Galactosyl-(1->3)-1D-myo-inositol, alpha-Maltose, beta-Maltose |
| 381.0795 | < 0.001 | 0.54 | C12H22O11 | [M+K]+ | 342.1162 | 0.28 |
| 383.0776 | < 0.001 | 0.57 | C12H22O11 | [M+(41K)]+ | 342.1162 | 0.18 |
| 401.1301 | < 0.001 | 0.41 | C12H22O11 | [M+Hac-H]- | 342.1162 | -0.02 | 2-alpha-D-Glucosyl-D-glucose, Cellobiose, D-Glucosyl-D-mannose, Epimelibiose, Inulobiose, Isomaltose, Lactose, Laminaribiose, Maltose, Melibiose, Nigerose, Palatinose, Sucrose, alpha,alpha-Trehalose, alpha-D-Galactosyl-(1->3)-1D-myo-inositol, alpha-Maltose, beta-D-Fructofuranosyl-alpha-D-mannopyranoside, beta-Maltose |
| 341.109 | < 0.001 | 0.46 | C12H22O11 | [M-H]- | 342.1162 | 0.09 |
| 379.0827 | 0.0144 | 0.55 | C12H22O11 | [M+(37Cl)]- | 342.1162 | 0.04 |
| 377.0857 | 0.0182 | 0.56 | C12H22O11 | [M+Cl]- | 342.1162 | 0.12 |
| 143.0349 | 0.0531 | 0.77 | C6H8O4 | [M-H]- | 144.0423 | -0.72 | 2,3-Dimethylmaleate, 2-Methyleneglutarate, Methylitaconate |
| 249.08 | < 0.0001 | 0.34 | C10H18O5S | [M-H]- | 250.0875 | -0.81 | 2-(5-Methylthio)pentylmalic acid, 3-(5-Methylthio)pentylmalic acid |
| 377.0857 | 0.0182 | 0.56 | C16H20O9 | [M+Na-2H]- | 356.1107 | 0.68 | 1-O-Feruloyl-beta-D-glucose |
| 563.1831 | < 0.001 | 0.43 | C18H32O16 | [M+Hac-H]- | 504.169 | 0.38 | 1F-beta-D-Fructosylsucrose, 6F-alpha-D-Galactosylsucrose, Cellotriose, D-Gal alpha 1->6D-Gal alpha 1->6D-Glucose, Raffinose |
| 527.1586 | < 0.001 | 0.48 | C18H32O16 | [M+Na]+ | 504.169 | 0.68 |
| 525.1442 | < 0.001 | 0.48 | C18H32O16 | [M+Na-2H]- | 504.169 | 0.86 |
| 543.1326 | < 0.001 | 0.53 | C18H32O16 | [M+K]+ | 504.169 | 0.76 |
| 503.1621 | < 0.001 | 0.54 | C18H32O16 | [M-H]- | 504.169 | 0.71 |
| 505.1767 | 0.0054 | 0.57 | C18H32O16 | [M+H]+ | 504.169 | 0.74 |
| 541.1359 | 0.0193 | 0.58 | C18H32O16 | [M+(37Cl)]- | 504.169 | 0.81 |
| 539.1388 | 0.0211 | 0.59 | C18H32O16 | [M+Cl]- | 504.169 | 0.61 |
| 209.0679 | 0.0088 | 0.76 | C8H10N4O3 | [M-H]- | 210.0753 | -0.5 | 1,3,7-Trimethyluric acid |
| 234.1287 | 0.0342 | 0.87 | C17H17N | [M-H]- | 235.1361 | -0.52 | (S)-7,8,13,14-Tetrahydroprotoberberine |
| 190.072 | < 0.001 | 0.29 | C5H9NO3 | [M+Hac-H]- | 131.0582 | -0.57 | (S)-4-Amino-5-oxopentanoate, 2-Amino-4-oxopentanoic acid, 4-Hydroxy-L-proline, 5-Amino-2-oxopentanoic acid, 5-Aminolevulinate, L-Glutamate 5-semialdehyde, N-Acetyl-beta-alanine, cis-4-Hydroxy-D-proline, trans-4-Hydroxy-L-proline, trans-L-3-Hydroxyproline |
| 172.0614 | 0.0026 | 0.61 | C5H7NO2 | [M+Hac-H]- | 113.0477 | -0.71 | (S)-1-Pyrroline-5-carboxylate, 1-Pyrroline-2-carboxylate, 3,4-Dihydro-2H-Pyrrole-2-carboxylate |
| 226.0486 | < 0.0001 | 0.33 | C8H15NO4 | [M+K-2H]- | 189.1001 | -0.51 | (2S)-2-{[1-(R)-Carboxyethyl]amino}pentanoate |
| 133.0971 | 0.0043 | 0.78 | C5H12N2O2 | [M+H]+ | 132.0899 | -0.11 | (2R,4S)-2,4-Diaminopentanoate, D-Ornithine, L-Ornithine, Ornithine |
| ^1^Fold change in intensity from long-day to short-day exposure groups. | | | | |  |  |  |

**Supplemental Table S3**. The eighteen metabolic KEGG pathways containing detected, significantly changing putative IDs ranked in order of raw, uncorrected p-value. The uncorrected p-value was used for this purpose as the majority of the FDR adjusted p-values equal 1, and hence cannot be ranked.

|  |  | **Input list** | | **Background** | | **Statistics** | |
| --- | --- | --- | --- | --- | --- | --- | --- |
| **Pathway name** | **Pathway Source** | **Hits** | **Total** | **Hits** | **Total** | ***p*-value** | **adjusted p-value** |
| D-Arginine and D-ornithine metabolism | KEGG | 7 | 200 | 7 | 442 | 0.023838 | 0.93933 |
| Starch and sucrose metabolism | KEGG | 15 | 200 | 18 | 442 | 0.024171 | 0.93933 |
| Amino sugar and nucleotide sugar metabolism | KEGG | 16 | 200 | 20 | 442 | 0.03796 | 0.93933 |
| Galactose metabolism | KEGG | 15 | 200 | 20 | 442 | 0.10109 | 1 |
| Phenylalanine metabolism | KEGG | 11 | 200 | 16 | 442 | 0.29468 | 1 |
| Purine metabolism | KEGG | 8 | 200 | 12 | 442 | 0.4077 | 1 |
| Alanine, aspartate and glutamate metabolism | KEGG | 6 | 200 | 9 | 442 | 0.45637 | 1 |
| Phenylalanine, tyrosine and tryptophan biosynthesis | KEGG | 8 | 200 | 14 | 442 | 0.6669 | 1 |
| Pyrimidine metabolism | KEGG | 9 | 200 | 17 | 442 | 0.78125 | 1 |
| Cysteine and methionine metabolism | KEGG | 9 | 200 | 17 | 442 | 0.78125 | 1 |
| Lysine degradation | KEGG | 6 | 200 | 12 | 442 | 0.82735 | 1 |
| beta-Alanine metabolism | KEGG | 6 | 200 | 13 | 442 | 0.8927 | 1 |
| Pentose and glucuronate interconversions | KEGG | 9 | 200 | 19 | 442 | 0.90199 | 1 |
| Glycine, serine and threonine metabolism | KEGG | 6 | 200 | 14 | 442 | 0.93573 | 1 |
| Tryptophan metabolism | KEGG | 9 | 200 | 21 | 442 | 0.96127 | 1 |
| Arginine and proline metabolism | KEGG | 16 | 200 | 35 | 442 | 0.96785 | 1 |
| Ascorbate and aldarate metabolism | KEGG | 7 | 200 | 19 | 442 | 0.98748 | 1 |
| Histidine metabolism | KEGG | 6 | 200 | 17 | 442 | 0.98849 | 1 |
